# Redox regulation of enzymes involved in sulfate assimilation and in the synthesis of sulfur-containing amino acids and glutathione in plants

**DOI:** 10.3389/fpls.2022.958490

**Published:** 2022-08-16

**Authors:** Linda de Bont, Natacha Donnay, Jérémy Couturier, Nicolas Rouhier

**Affiliations:** ^1^Université de Lorraine, INRAE, IAM, F-54000, Nancy, France; ^2^Institut Universitaire de France, F-75000, Paris, France

**Keywords:** cysteine, methionine, redox, sulfate, plants, post-translational modifications, glutathione

## Abstract

Sulfur is essential in plants because of its presence in numerous molecules including the two amino acids, cysteine, and methionine. Cysteine serves also for the synthesis of glutathione and provides sulfur to many other molecules including protein cofactors or vitamins. Plants absorb sulfate from their environment and assimilate it *via* a reductive pathway which involves, respectively, a series of transporters and enzymes belonging to multigenic families. A tight control is needed to adjust each enzymatic step to the cellular requirements because the whole pathway consumes energy and produces toxic/reactive compounds, notably sulfite and sulfide. Glutathione is known to regulate the activity of some intermediate enzymes. In particular, it provides electrons to adenosine 5′-phosphosulfate reductases but also regulates the activity of glutamate-cysteine ligase by reducing a regulatory disulfide. Recent proteomic data suggest a more extended post-translational redox control of the sulfate assimilation pathway enzymes and of some associated reactions, including the synthesis of both sulfur-containing amino acids, cysteine and methionine, and of glutathione. We have summarized in this review the known oxidative modifications affecting cysteine residues of the enzymes involved. In particular, a prominent regulatory role of protein persulfidation seems apparent, perhaps because sulfide produced by this pathway may react with oxidized thiol groups. However, the effect of persulfidation has almost not yet been explored.

## Introduction

**FIGURE 1:**
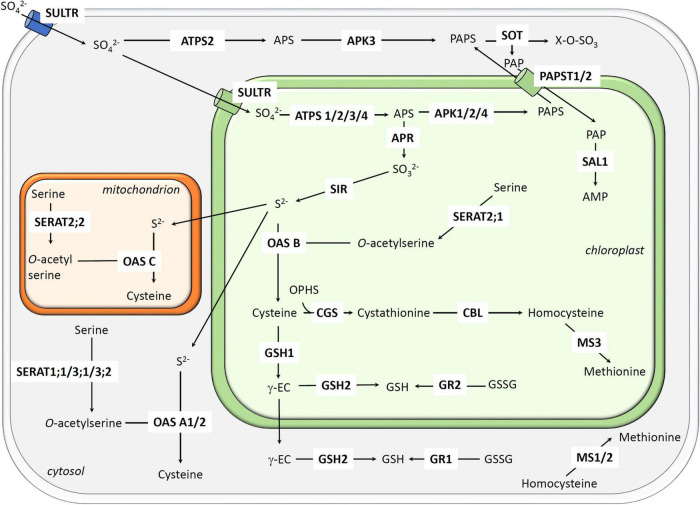
Subcellular localizations and reactions catalyzed by enzymes involved in sulfate assimilation and cysteine, methionine, and glutathione synthesis. Abbreviations of proteins (in bold) are as follows: ATPS, ATP-sulfurylase; APK, APS kinase; APR, APS reductase; CBL, cystathionine β-lyase; CGS, cystathionine γ-synthase; GSH1, γ-glutamate cysteine ligase; GSH2, glutathione synthetase; GR, glutathione reductase; MS, methionine synthase; OAS, o-acetylserine (thiol) lyase; PAPST, phosphosulfate transporter; SERAT, serine acetyltransferase; SIR, sulfite reductase; SOT, sulfotransferase; SULTR; sulfate transporter. Abbreviations of metabolites are as follows: AMP, adenosine monophosphate; APS, adenosine 5′-phosphosulfate; OPHS, *o*-phosphohomoserine; PAP, 3′-phosphoadenosine 5′-phosphate; PAPS, 3′-phosphoadenosine 5′-phosphosulfate; SO_4_^2–^: sulfate, SO_3_^2–^: sulfite, S_2_^–^: sulfide; γ-EC, γ-glutamyl cysteine; X-O-SO_3_: sulfated compound.

Sulfate is the major source of sulfur for both aquatic and terrestrial plants (Takahashi et al., 2011). After absorption, the first step of the reductive assimilation of sulfate is its adenylation to adenosine 5′-phosphosulfate (APS), a reaction catalyzed by ATP sulfurylases (ATPS) (Figure 1; Bohrer and Takahashi, 2016). In a primary sulfur metabolic pathway, APS is reduced by APS reductases (APR) which form sulfite (SO_3_^2–^) at the expense of glutathione. Sulfite is then reduced to sulfide by sulfite reductase (SIR) (Takahashi et al., 2011). In the next step, the O-acetyl-serine thiol-lyases (OAS-TL) promote the formation of cysteine by incorporating sulfide into O-acetyl-serine formed by serine acetyltransferases (SERAT) from serine. Both OAS-TL and SERAT are part of the cysteine synthase complex (Wirtz et al., 2010). In addition to be part of proteins, an important source of cysteine consumption is the synthesis of methionine and glutathione. The two-step synthesis of the glutathione tripeptide requires two ATP-dependent enzymes, namely glutamate cysteine ligase (GCL/γ-ECS/CAD2/GSH1) and glutathione synthetase (GSH2) (Müller-Schüssele et al., 2020). Methionine biosynthesis occurs in three consecutive reactions catalyzed by cystathionine γ-synthase (CGS), cystathionine β-lyase (CBL), and methionine synthases (MS) (Ravanel et al., 1998b). In a secondary sulfur metabolic pathway, APS phosphorylation catalyzed by APS kinases (APK) forms phosphoadenosine 5′-phosphosulfate (PAPS) (Takahashi et al., 2011). PAPS provides sulfur for the synthesis or modification of several secondary metabolites including glucosinolates, peptides or hormones in reactions involving sulfotransferases (SOT). Sulfate conjugation reactions catalyzed by SOT generate 3′-phosphoadenosine 5′-phosphate (PAP), a molecule that participates in the organelle-nuclear retrograde signaling, if not dephosphorylated by the SAL1 phosphatase (Estavillo et al., 2011).

While many essential steps of the sulfate assimilation and cysteine, methionine and glutathione biosynthesis pathways occur exclusively in plastids, a few enzymes present in the cytosol and mitochondria also come into play, which necessitates a concerted action between enzymes present in different subcellular compartments and intracellular transporters. Moreover, it is critical to coordinate the sulfate assimilation pathway with the ones for nitrogen assimilation and carbon dioxide fixation, notably for the synthesis of amino acids. For this reason, sulfate assimilation, which is highly regulated by the demand for reduced sulfur, is also notably dependent on the nitrogen status (Kopriva and Koprivova, 2004). In fact, many genes coding for sulfate transporters and for enzymes of the primary assimilation pathway are induced at the transcriptional level by sulfur deficiency whereas reduced sulfur-containing compounds, such as hydrogen sulfide (H_2_S), cysteine, S-adenosyl-methionine (SAM) and glutathione, exert inhibitory effects at the post-translational level (Kopriva and Koprivova, 2004; Galili et al., 2016). The inhibition by glutathione was for instance demonstrated in the case of ATPS, APR, and GCL. Still, the major regulatory steps of the sulfate assimilation pathway are at the APR and cysteine synthase level. Acting at these steps should indeed avoid the accumulation of sulfite and sulfide, which may otherwise be toxic. As a matter of fact, APR gene expression or enzyme activity is regulated by many conditions or treatments in which other genes/enzymes are not regulated. As the rate-limiting step, it was noticed that regulation of APR is a good approximation of regulation of the whole sulfate assimilation pathway (Kopriva, 2006). Concerning the cysteine synthase complex, regulation occurs notably at the post-translational level to coordinate the action of SERAT and OAS-TL depending on the availability of the respective substrates, sulfide or OAS (Wirtz et al., 2010). Another layer of regulation that occurs at the post-translational level is the modification of critical and reactive cysteine residues. This represents a rapid, efficient and reversible way to shut down or activate protein activity/function (Couturier et al., 2013). The thiol group of cysteines reacts with a diverse array of molecules referred to as reactive oxygen, nitrogen, sulfur, or carbonyl species, generating various types of redox post-translational modifications (PTMs) (Couturier et al., 2013). In addition to regulatory purposes, redox PTMs that prevent the irreversible oxidation of cysteine residues may be seen as a protective mechanism. This is particularly true in the context of stress conditions that generate peroxides. Indeed, by reacting with peroxides, cysteines will be progressively oxidized to sulfenic acid state (SOH), to the partially reversible sulfinic acid state (SO_2_H) and to the irreversible sulfonic acid state (SO_3_H). It is commonly accepted that reaction of sulfenic acids with reduced glutathione (GSH) or H_2_S prevents cysteine overoxidation. This leads respectively to the formation of a glutathione adduct (S-SG) and of a persulfide group (S-SH), two reversible modifications referred to as glutathionylation and persulfidation. Noteworthy, other biochemical reactions lead to glutathionylation and persulfidation (Zaffagnini et al., 2012b; Filipovic et al., 2018). Nitrosylation of cysteine residues is another important redox PTM and nitrosoglutathione (GSNO) may have a prominent role in plant cells. The reverse reactions, i.e., reductions, are catalyzed by thiol-disulfide oxidoreductases belonging to the thioredoxin (TRX) superfamily. In particular, deglutathionylation is mainly catalyzed by glutaredoxins (GRX), denitrosylation by GSH and TRX to some extent, whereas both GRX and TRX seem to be efficient for depersulfidation reactions in addition to disulfide bond reduction (Rouhier et al., 2008; Zaffagnini et al., 2016; Moseler et al., 2021). In this review, we discuss the current knowledge about how the oxidative modifications of protein thiol groups of the different enzymes involved in sulfate assimilation pathway and in cysteine, methionine, and glutathione biosynthesis regulate sulfur metabolism in plants and provide perspectives about new levels of redox regulation based on proteomics data (Supplementary Table 1).

## Redox control of cysteine biosynthesis

### Redox regulation at the level of the first step catalyzed by ATP sulfurylases

In *Arabidopsis thaliana*, four genes encode plastid-located ATP sulfurylases (ATPS1–4) with ATPS2 being additionally expressed in the cytosol (Bohrer et al., 2015). There is an important diversity in terms of domain organization among members of this family but ATPS from higher plants are formed by a single ATPS domain and exist as homodimers (Herrmann et al., 2015). Noteworthy, chimeric enzymes in which the ATPS domain is fused with an APS kinase domain exist in human, fungi and some diatoms (Harjes et al., 2005; Prioretti et al., 2014).

**FIGURE 2:**
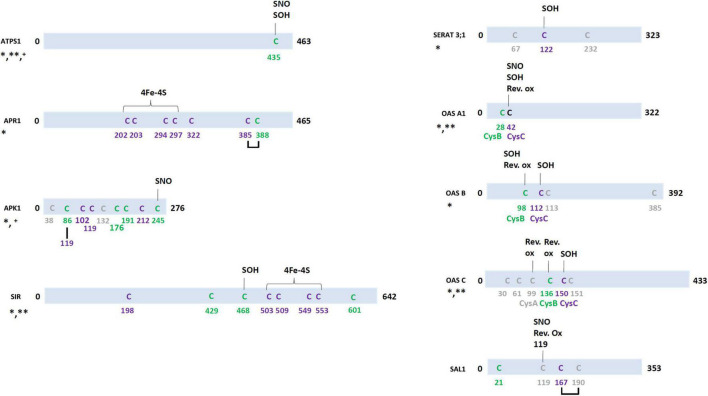
Redox post-translational modifications of enzymes involved in sulfate assimilation pathway in the green lineage. In purple, cysteines conserved in all Arabidopsis isoforms and in at least one isoform from *Chlamydomonas reinhardtii* (green alga), *Physcomitrella patens* (Bryophyte), *Selaginella moellendorffii* (Lycophyte), *Oryza sativa* (Monocot), *Zea mays* (Monocot), *Glycine max* (Annual eudicot), *Brassica oleracea* (Biennal eudicot), *Solanum lycopersicum* (Perennial), *Populus trichocarpa* (Perennial), and *Vitis vinifera* (Perennial). In green, cysteines conserved in all Arabidopsis isoforms and at least one isoform from other terrestrial plants. In gray, cysteines present in *A. thaliana* isoform but not conserved in isoforms from other plants. For nitrosylation (SNO), reversible cysteine oxidation (Rev. ox.) and sulfenylation (SOH), targeted cysteines are known and they are indicated on the scheme. When targeted cysteines are unknown, the following code applies: *, persulfidation; **, TRX target; ^+^, glutathionylation. For OAS-TL isoforms, the nomenclature CysA, B and C refers to the text. The scheme is based on the indicated *A. thaliana* isoforms, but additional information for missing isoforms in multigene families are found in Supplementary Tables 2,3.

Concerning the regulation of the expression and activity of ATPS in plants, it was reported that ATPS activity is increased by sulfur deprivation and H_2_O_2_ treatment in *Brassica napus* roots but inhibited by GSH (Lappartient and Touraine, 1996, 1997; Lappartient et al., 1999). Although ATPS isoforms from terrestrial plants are rather distant from those present in algae and cyanobacteria and constitute an independent phylogenetic group, additional evidence for a possible redox control of ATPS activity come from analyses performed with ATPS from algae and cyanobacteria. Two phylogenetic clades/classes have been defined in these organisms (Patron et al., 2008; Prioretti et al., 2016). A treatment with reduced DTT increased activity of the previously defined class B ATPS, which are characterized by the presence of five conserved cysteines, but not of ATPS from the A class despite they also contain conserved cysteine residues but at different positions (Prioretti et al., 2014, 2016). Interestingly, large-scale redox proteomic analyses performed in the cyanobacterium *Synechocystis* sp. showed that ATPS is glutathionylated and retained on a TRX affinity column (Lindahl and Florencio, 2003; Chardonnet et al., 2015). Similar experiments performed in the alga *Chlamydomonas reinhardtii* reported that Cys261 of ATS1 could be either nitrosylated or glutathionylated (Figure 2 and Supplementary Table 2; Zaffagnini et al., 2012a; Morisse et al., 2014). The effect of these modifications on protein activity is unknown, but this cysteine is also present in *C. reinhardtii* ATS2 and other microalgal orthologs. The corresponding cysteine in ATPS of the diatom *Phaeodactylum tricornutum* has been described to be sensitive to oxidation by H_2_O_2_, as well as several other less conserved cysteine residues (Rosenwasser et al., 2014). While higher plant ATPS usually have a single cysteine residue in the C-terminal region (Cys435 in AtATPS1) which is absent in algal sequences, similar redox PTMs have been reported for *A. thaliana* ATPS. AtATPS1 was found to be prone to sulfenylation and nitrosylation (Hu et al., 2015; Wei et al., 2020) and AtATPS2 to glutathionylation (Dixon et al., 2005). Moreover, ATPS1, 2, and 4 have been reported as persulfidated (Jurado-Flores et al., 2021). All these modifications may explain why AtATPS1 was isolated as a TRX target in Arabidopsis leaves (Marchand et al., 2006). From the 3D structure of *Glycine max* ATPS1, the cysteine found at position 434 (corresponding to Cys435 in AtATPS1) seems exposed at the surface, but the effect of these different modifications on protein structure or activity is yet unexplored (Herrmann et al., 2015).

### Redox control at the adenosine 5′-phosphosulfate branching point

In the chloroplasts, APS can either be reduced to sulfite by APR or be phosphorylated to PAPS by APK. The partitioning of the flux at this step is clearly important for the plant sulfur metabolism. A sulfate deprivation favors APS reduction for promoting cysteine, methionine, and glutathione synthesis whereas the presence of reduced sulfur sources represses APS reduction in the favor of APS phosphorylation (Takahashi et al., 1997; Kopriva et al., 2012). To modulate this channeling, redox switches occur for both APR and APK enzymatic activities (Jez et al., 2016).

In *A. thaliana*, the APR family is formed by three genes coding for chloroplastic proteins. Higher plant APR are homodimers with each monomer formed by an N-terminal domain binding a [4Fe-4S] cluster and bearing the APR activity and a C-terminal GRX-related domain (Bick et al., 1998; Kopriva et al., 2002). They usually contain seven conserved Cys residues, five in the N-terminal domain which include four iron-sulfur (Fe-S) cluster ligands and a catalytic residue and two in the GRX-like domain (Figure 2 and Supplementary Table 2). While the Fe-S cluster is critical for activity, its role for the reaction mechanism remains unclear (Carroll et al., 2005). Other cysteines are mandatory as well. Indeed, the first catalytic step involves the transfer of sulfate from APS to the active cysteine residue (Cys322 in AtAPR1) forming a covalent S-sulfo-cysteine intermediate. Sulfite is then released upon reduction of this intermediate that is mediated by GSH and the cysteines of the CxxC motif (Cys385 and 388 in AtAPR1) present in the C-terminal GRX-like domain. Whether the first nucleophilic attack is performed by GSH or the first cysteine residue of the CxxC motif remains debated. In the light of this proposed catalytic mechanism, some previous results about redox modulation of APR seem counterintuitive. Indeed the catalytic efficiency of APR was described as activated by oxidation, either *in vitro* using glutathione disulfide (GSSG) or *in vivo* in response to ozone and paraquat, and inactivated upon reduction (Bick et al., 2001). Based on gel filtration analysis and site-directed mutagenesis, it was suggested that a disulfide bond joined two monomers at least in AtAPR1 (Kopriva and Koprivova, 2004). However, recombinant proteins used were often devoid of the Fe-S cluster which is problematic because four cysteine residues now become possibly reactive and blur the conclusions. Hence, besides biochemical evidence for the reduction of the intramolecular disulfide formed in the C-terminal domain of APR by GSH, additional evidence for other redox regulatory mechanisms remain scarce. Consistently, redox proteomics studies did not point to much redox PTMs except that AtAPR1, 2, and 3 could become persulfidated under S-sufficient media (Aroca et al., 2017; Jurado-Flores et al., 2021).

Four APK proteins exist in Arabidopsis, APK1, 2, and 4 locate in plastids whereas APK3 is in the cytosol (Mugford et al., 2009). APK activity is repressed under sulfur-deficient conditions (Maruyama-Nakashita et al., 2006). AtAPK1 is a homodimeric enzyme both in the reduced and oxidized form but the catalytic efficiency of the reduced form is enhanced by a factor 17 (Ravilious et al., 2012; Jez et al., 2016). An intermolecular disulfide bond formed between Cys86 and Cys119 was visible in AtAPK1 structure (Ravilious et al., 2012). The redox potential (E_*m*_ value around – 250 mV at pH 7) is consistent with the observed reduction by GSH. It was proposed that disulfide formation alters catalytic efficiency by affecting the dynamic movements of the N-terminal loop (Ravilious et al., 2012). Both cysteines are conserved in most chloroplastic orthologs from terrestrial plants whereas only Cys119 is present in the cytosolic AtAPK3 or in algal representatives (Figure 2 and Supplementary Table 3). This might not be the only redox switch for this protein since four other cysteine residues are well conserved in APKs from terrestrial plants. While the targeted residue(s) is(are) unknown, AtAPK1 and AtAPK4 are likely subject to persulfidation (Aroca et al., 2017; Jurado-Flores et al., 2021). Moreover, a partially conserved cysteine (Cys245 in AtAPK1) was found nitrosylated (Hu et al., 2015). The activity of a rice APK, but not of variants mutated for the corresponding Cys86 and Cys119 of AtAPK1, is decreased *in vitro* in the presence of GSSG, suggesting protein glutathionylation (De-zhen et al., 2016). In accordance, *C. reinhardtii* APK was detected in a proteomic study focusing on glutathionylation (Zaffagnini et al., 2012a). This suggests that glutathionylation of one of these cysteine residues may be an intermediate toward the formation of the intermolecular disulfide in isoforms possessing both cysteines. Overall, these described redox PTMs may explain why *C. reinhardtii* APK was identified as a TRX-target as well (Pérez-Pérez et al., 2017).

### Redox regulation at the level of the secondary sulfur metabolic pathway

While SOT represent the largest protein family among sulfate-assimilating proteins, their biochemical and functional characterization still lags behind and this is also true for the associated regulation mechanisms. Among the 18 Arabidopsis SOT, there are two conserved cysteines (positions 199 and 270, AtSOT1 numbering) (Klein and Papenbrock, 2004). Based on an AtSOT1 structural model generated by alphafold, both cysteines are too far away for predicting any redox regulation mechanism involving an intramolecular disulfide. However, unlike Cys270, Cys199 is surface-exposed, which may fit the observation that about half of the SOT were retrieved in the recent persulfidome analyses. So far, a possible effect of these modifications remains unclear.

The SAL1 phosphatase, which is located both in chloroplasts and mitochondria, regulates PAP levels by dephosphorylating it to AMP. In *A. thaliana*, PAP accumulates notably in response to drought and high light stresses. It was shown that the SAL1-PAP pathway is important for the organelle-nuclear retrograde signaling in this context. The redox inactivation of SAL1 promotes the transport of PAP from chloroplasts (and mitochondria) *via* PAPST transporters to the nucleus where it inhibits exoribonuclease-mediated RNA metabolism (Estavillo et al., 2011; Chan et al., 2016). This allows the transcriptional reprogramming necessary for stress tolerance. The Arabidopsis SAL1 possesses four cysteines at positions 21, 119, 167, and 190 (Figure 2 and Supplementary Table 3). Under oxidative stress conditions such as high light or drought, AtSAL1 is partially inactivated in a redox-dependent manner. Using SAL1 recombinant protein treated with reduced or oxidized forms of DTT and glutathione, it appeared that Cys119 is involved in an intermolecular disulfide bridge that led to the formation of a dimeric form and that this would favor the formation of an additional intramolecular disulfide bridge between Cys167 and Cys190 (Chan et al., 2016). *In vitro* glutathionylation with GSSG leads to glutathionylation of Cys119 and to the formation of the Cys167–Cys190 intramolecular disulfide (Chan et al., 2016). Unlike Cys119, both Cys167 and Cys190 are conserved in terrestrial plants, although Cys190 is shifted by 7 amino acids in monocots. Other interesting information are the observation that SAL1 is also subject to persulfidation (Aroca et al., 2017; Jurado-Flores et al., 2021) and to S-nitrosylation of Cys119 which is adjacent to an Asp residue (Asp118), a combination that is particularly suitable for S-nitrosylation (Hu et al., 2015).

### Redox regulation at the level of the primary sulfur metabolic pathway

Sulfite reductase is a 70 kDa enzyme that catalyzes the reduction of sulfite into sulfide. It binds a [4Fe-4S] cluster covalently linked to a siroheme as redox centers and uses ferredoxin as an electron donor. In Arabidopsis, SIR is encoded by a single, essential gene and is described as a “bottleneck” in the sulfate assimilation pathway (Khan et al., 2010). In order to protect cells against sulfite toxicity, *SIR* expression is rapidly upregulated in Arabidopsis and tomato in response to higher sulfite content, and accordingly SIR activity increases (Yarmolinsky et al., 2013). *SIR* transcripts are also upregulated after cold stress and methyl viologen treatment in maize (Xia et al., 2018). Using proteomic approaches, SIR has been identified as a TRX target in wheat seeds (Wong et al., 2004) and in *Synechocystis* (Lindahl and Florencio, 2003). Five cysteine residues are conserved in SIR sequences and four of them serve as Fe-S cluster ligands. In fact, an additional partially conserved Cys468 is targeted by sulfenylation (Wei et al., 2020). As for most other enzymes of the pathway, SIR may be persulfidated, but neither the cysteine affected nor the effect on activity have been explored (Figure 2 and Supplementary Table 2).

Cysteine synthesis takes place in plastids, cytosol, and mitochondria with the combined action of OAS-TL and SERAT isoforms that form a hetero-oligomeric complex referred to as cysteine synthase (Wirtz et al., 2010). In Arabidopsis, there are four OAS-TL isoforms: OAS A1, A2, B, and C (Jost et al., 2000), the cytosolic isoform OAS A1 being the main contributor for cysteine biosynthesis (López-Martín et al., 2008). The proteins have been identified in numerous redox proteomic data as having a quite large variety of redox PTMs. Both OAS A1 and OAS C have been described as targets of TRX (Marchand et al., 2006; Yoshida et al., 2013), OAS C as a target of GRX (Rouhier et al., 2005), while OAS A1, OAS B, and OAS C have been reported as being persulfidated (Jurado-Flores et al., 2021). In these cases, there is no information about the cysteines involved. Other reported modifications occur on three cysteines. Because these cysteines have very different numbering depending on the isoform, we refer to CysA for the cysteine that is present only in the N-terminal part of organellar OAS-TL (OAS B and OAS C), whereas CysB and CysC represent the two cysteine residues that are conserved in all Arabidopsis OAS-TL. In OAS A1 and OAS A2, only CysC (Cys42) may be oxidized both by nitrosylation and sulfenylation (Figure 2 and Supplementary Table 2; Hu et al., 2015; Wei et al., 2020). In OAS B, CysB (Cys98), and CysC (Cys112) could be sulfenylated (Huang et al., 2019; Wei et al., 2020). In OAS C, the three cysteines may be subject to redox PTM. CysA (Cys99) and CysB (Cys136) can be reversibly oxidized but the modification is unknown (Liu et al., 2014; Nietzel et al., 2020) whereas CysC (Cys150) could be sulfenylated (Wei et al., 2020). The fact that CysC is sulfenylated in all OAS together with the observed persulfidation of three OAS isoforms may suggest a two-step mechanism in which sulfenylation precedes persulfidation, i.e., when sulfenic acids react with H_2_S.

Five genes encode SERAT in *A. thaliana*. SERAT1;1, -3;1, and -3;2 locate in the cytosol, SERAT2;1 in chloroplasts and SERAT2;2 in mitochondria (Kawashima et al., 2005; Watanabe et al., 2018). The mitochondrial SERAT2;2 is responsible of 80% of SERAT activity (Watanabe et al., 2008). SERAT3;1 and -3;2 are unable to bind OAS-TL (Kawashima et al., 2005). SERAT2;1 is also part of a chloroplastic complex named COPS (containing cyclophilin 20-3, OAS B, 2-cysteine peroxiredoxins A/B, and SERAT2;1). The action of this COPS module, involved in plant acclimation to high light stress, is influenced by redox stimuli and oxylipin signaling (Müller et al., 2017). A feedback inhibition by cysteine decreased SERAT1;1 and -3;1 activity but appeared to have no impact on the three other SERAT isoforms (Kawashima et al., 2005). In fact, these two isoforms, as well as SERAT 2;2, have been reported to be persulfidated (Jurado-Flores et al., 2021). All Arabidopsis SERAT, except SERAT1;1, have been detected in sulfenylomes (Figure 2 and Supplementary Table 2; Huang et al., 2019; Wei et al., 2020). AtSERAT2;1, -3;1, and -3;2 are sulfenylated on the same conserved cysteine (Cys150 in AtSERAT2;1, Cys122 in AtSERAT3;1, and Cys159 in AtSERAT3;2), whereas AtSERAT2;2 is sulfenylated on two cysteines that are not conserved among Arabidopsis SERAT. As for OAS-TL, it may be that sulfenylation and persulfidation of SERAT are intimately connected.

## Redox control of glutathione biosynthesis and reduction

**FIGURE 3:**
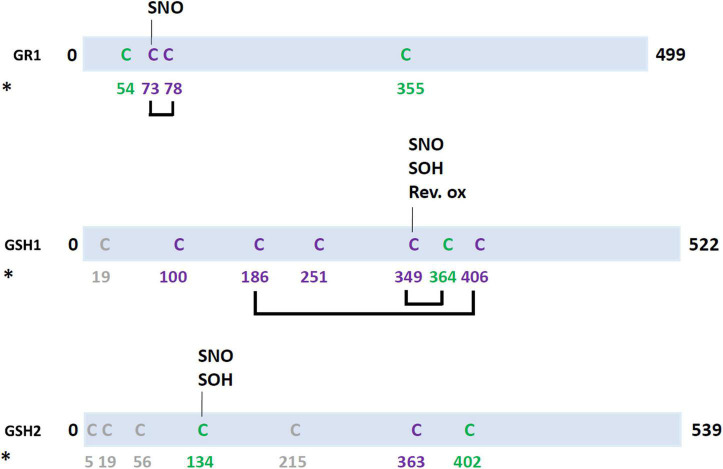
Redox post-translational modifications of enzymes involved in glutathione biosynthesis pathway in the green lineage. In purple, cysteines conserved in all Arabidopsis isoforms and in at least one isoform from *Chlamydomonas reinhardtii* (green alga), *Physcomitrella patens* (Bryophyte), *Selaginella moellendorffii* (Lycophyte), *Oryza sativa* (Monocot), *Zea mays* (Monocot), *Glycine max* (Annual eudicot), *Brassica oleracea* (Biennal eudicot), *Solanum lycopersicum* (Perennial), *Populus trichocarpa* (Perennial), and *Vitis vinifera* (Perennial). In green, cysteines conserved in all Arabidopsis isoforms and at least one isoform from other terrestrial plants. In gray, cysteines present in *A. thaliana* isoform but not conserved in isoforms from other plants. For nitrosylation (SNO), reversible cysteine oxidation (Rev. ox.) and sulfenylation (SOH), targeted cysteines are known and they are indicated on the scheme. When targeted cysteines are unknown, the following code applies: *, persulfidation.

Glutathione synthesis occurs in two steps. GCL/GSH1 is exclusively located in plastids in higher plants and encoded by a single gene in *A. thaliana* whereas *A. thaliana* glutathione synthetase (GS/GSH2) is expressed in both plastids and cytosol (Wachter et al., 2005). Currently, it is documented that the major redox control for these enzymes is a feedback inhibition exerted by GSH on GCL (Meyer and Fricker, 2002). Even though GSH2-catalyzed reaction is not considered as a limiting step, it may be regulated in some specific context and organisms. Indeed, *A. thaliana* GSH2 is the target of several redox PTMs that could impact its activity and thus modulate glutathione biosynthesis, i.e., sulfenylation (Wei et al., 2020) and nitrosylation (Hu et al., 2015) of Cys134 and persulfidation (Figure 3 and Supplementary Table 4; Aroca et al., 2017; Jurado-Flores et al., 2021). However, Cys134 is only partially conserved and there is actually no strictly conserved cysteine in GSH2 from terrestrial plants. Moreover, the activity of GSH2 in roots of Arabidopsis seedlings treated with H_2_O_2_ was not affected, unlike GCL (Hicks et al., 2007).

Concerning GCL, while two disulfides bonds (Cys186–Cys406 and Cys349–Cys364, Arabidopsis GCL numbering) are present in orthologs from Rosids, only the first one is involved in the redox modulation of the enzyme activity (Hothorn et al., 2006; Hicks et al., 2007; Gromes et al., 2008). The two additional cysteine residues present in *A. thaliana* GCL do not seem to be oxidatively modified despite being surface-exposed. In fact, among these six cysteines, only one (Cys349) was identified as a target of nitrosylation (Hu et al., 2015) and sulfenylation (Wei et al., 2020). A possible persulfidation is also reported but the targeted cysteine(s) not identified (Aroca et al., 2017; Jurado-Flores et al., 2021). Noticeably, the Cys364 is not conserved in GCL from several plant species. In terms of redox control, reduction of the Cys186-Cys406 disulfide bridge considerably decreases protein activity (Hicks et al., 2007; Gromes et al., 2008). Oxidized GCLs form homodimers, both *in vitro* and *in vivo*, which dissociate into monomers upon reduction (Hothorn et al., 2006; Gromes et al., 2008; Yang et al., 2019). However, using variants that do not dimerize, it has been recently described that homodimer formation of GCL is not required for enzyme activation (Yang et al., 2019). Intriguingly, Cys186 and Cys406 are not present in GCL sequences from some green algae (Gromes et al., 2008). Moreover, despite possessing the residue corresponding to Cys186 and Cys406, some phylogenetically related proteobacterial GCLs do not dimerize upon oxidation and their activity is not impacted by a treatment with reduced DTT (Gromes et al., 2008). In conclusion, this negative feedback loop associated with the above-described thiol-based switch mechanism seems to have been evolutionary favored by the confinement of GSH biosynthesis and of the GCL enzyme in the plastidial compartment of some photosynthetic organisms but not all (or get lost or replaced in some of them).

The reduction of GSSG into GSH is catalyzed by glutathione reductases (GR) using NADPH as an electron donor. Two GR genes are present in *A. thaliana*. GR1 is present in the cytosol and peroxisomes and GR2 in mitochondria and chloroplasts (Müller-Schüssele et al., 2020). In Arabidopsis, only the plastidial GR2 is essential (Marty et al., 2019). This is likely due to the existence of intracellular GSH transporters and of some alternative back-up reducing systems. Not much is known about a possible redox control at this step and the reason may be that an efficient GSH reduction system is constitutively needed. A GR from rice was reported as being *S*-nitrosylated in a catalase mutant (*noe1*) which displays elevated level of H_2_O_2_ that promotes NO production (Lin et al., 2012). Moreover, pea chloroplastic and cytosolic GRs can be S-nitrosylated *in vitro* by GSNO, but it did not impact their activity (Begara-Morales et al., 2015). The poplar GR2 was retained on an affinity column with a bound poplar GRX C4 C30S variant (Rouhier et al., 2005). Arabidopsis GR1 and GR2 possess 8 and 9 cysteines respectively but only the two catalytic cysteines at position 73 and 78 in AtGR1 are strictly conserved and Cys73 was reported as being subject to nitrosylation (Figure 3 and Supplementary Table 4; Hu et al., 2015). The reason why there might be no effect on GR activity is that the nitrosylated cysteine could be resolved into a regular disulfide by the second cysteine. The AtGR1 and AtGR2 isoforms may also be subject to cysteine persulfidation both in leaves and roots (Aroca et al., 2017; Jurado-Flores et al., 2021). GR1 and GR2 have both been identified in roots but only GR1 displays more persulfidation in a N-starved medium compared to a N-sufficient medium.

## Redox control at the level of methionine biosynthesis

Enzymes catalyzing the two first steps of methionine synthesis, i.e., CGS and CBL, are present only in chloroplasts and are encoded by a single gene while MS are encoded by three genes, with two isoforms being expressed in the cytosol (MS1 and MS2) and one in chloroplasts (MS3) (Figure 1; Ravanel et al., 1998b). The chloroplastic isoform is likely required for the *de novo* methionine synthesis whereas cytosolic isoforms are most probably involved in the regeneration of Met from homocysteine (Ravanel et al., 2004).

**FIGURE 4:**
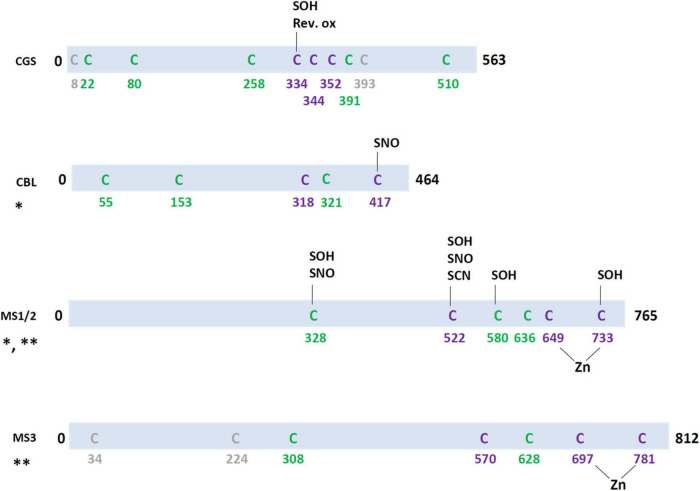
Redox post-translational modifications of enzymes involved in methionine biosynthesis pathway in the green lineage. In purple, cysteines conserved in all Arabidopsis isoforms and in at least one isoform from *Chlamydomonas reinhardtii* (green alga), *Physcomitrella patens* (Bryophyte), *Selaginella moellendorffii* (Lycophyte), *Oryza sativa* (Monocot), *Zea mays* (Monocot), *Glycine max* (Annual eudicot), *Brassica oleracea* (Biennal eudicot), *Solanum lycopersicum* (Perennial), *Populus trichocarpa* (Perennial), and *Vitis vinifera* (Perennial). In green, cysteines conserved in all Arabidopsis isoforms and at least one isoform from other terrestrial plants. In gray, cysteines present in *A. thaliana* isoform but not conserved in isoforms from other plants. For nitrosylation (SNO), reversible cysteine oxidation (Rev. ox.), S-cyanylation (SCN) and sulfenylation (SOH), targeted cysteines are known and they are indicated on the scheme. When targeted cysteines are unknown, the following code applies: *, persulfidation; **, TRX target.

The CGS is a pyridoxal 5′-phosphate (PLP)-containing homo-tetramer that uses *o*-phosphohomoserine (derived from aspartate) and cysteine as substrates for cystathionine biosynthesis (Ravanel et al., 1998a). Compared with bacterial CGS, plant CGS have an N-terminal extension (amino acids 77-87) that displays a regulatory role (Hacham et al., 2002). While it does not seem required for its enzymatic activity, overexpression of a truncated version of Arabidopsis CGS that lacks this N-terminal region in *Nicotiana tabacum* transgenic plants highlighted both a strongly altered development and an impaired Met metabolism. The authors concluded that PTMs may occur at the level of this N-terminal region. The regulatory role of the N-terminal region of CGS has also been described upon induction of folate deficiency in Arabidopsis (Loizeau et al., 2007). Regarding possible thiol regulatory switches happening at this step, it is worth noting that *A. thaliana* CGS displays eight cysteines in its mature part (two cysteines are in the chloroplastic targeting sequence) but only three are conserved in enzymes of the green lineage (Cys334, Cys344, and Cys352) (Figure 4 and Supplementary Table 5). So far, only Cys334 was identified as subject to reversible oxidation and as a target of sulfenylation (Liu et al., 2014; Wei et al., 2020). It is now needed to study the impact of these modifications on protein activity.

The CBL primarily catalyzes the cleavage of cystathionine, producing homocysteine, pyruvate, and ammonia. Similar to CGS, *A. thaliana* CBL is a PLP-dependent homo-tetramer (Ravanel et al., 1996). Concerning redox PTMs, one of the two cysteines conserved in CBLs from the green lineage analyzed is prone to nitrosylation (Cys417) (Figure 4 and Supplementary Table 5; Hu et al., 2015). The protein has been also found to be targeted by persulfidation but the cysteine is unknown (Jurado-Flores et al., 2021). Nevertheless, if any, the regulatory role of these PTMs has not been described yet.

Methionine synthases catalyze the final reaction in the methionine biosynthesis pathway, i.e., the transfer of a methyl group from 5-methyltetrahydrofolate to homocysteine, thus generating tetrahydrofolate and methionine. *A. thaliana* possesses three genes, At5g17920, At3g03780, and At5g20980 respectively named MS1, MS2, and MS3. These are all cobalamin- or vitamin B_12_-independent enzymes. The structural analysis of the monomeric *A. thaliana* MS1 indicates that two cysteines (Cys649 and Cys733) and one histidine (His647) bind a zinc atom that is required for activity (Ferrer et al., 2004). The *A. thaliana* MS isoforms have a variable number of cysteine residues, but only three of them are conserved, including the two zinc-binding cysteines (Figure 4 and Supplementary Table 5). Unlike, the *C. reinhardtii* ortholog of Arabidopsis MS3 which was detected as glutathionylated or nitrosylated (Zaffagnini et al., 2012a; Morisse et al., 2014), no redox PTM was identified for the chloroplastic Arabidopsis MS3 itself in such specific proteomic studies. Still, it was identified as TRX or GRX targets (Wong et al., 2004; Rouhier et al., 2005). The cytosolic MS1 was also immobilized on an affinity column grafted with a variant for a cytosolic TRX h (Yamazaki et al., 2004). Both cytosolic and plastidial MS from *C. reinhardtii* were also identified as TRX partners (Pérez-Pérez et al., 2017). In addition, four cysteines of AtMS1 and AtMS2, not all conserved but including the zinc-binding Cys733, have been described to be subject to sulfenylation (Huang et al., 2019; Wei et al., 2020), two of them to nitrosylation (Hu et al., 2015) and one to S-cyanylation (García et al., 2019). The latter modification was detected in extracts from an *A. thaliana* mutant for a mitochondrial β-cyanoalanine synthase (CAS-C1) that is normally able to detoxify hydrogen cyanide. In conclusion, it seems that the redox control exerted on the methionine synthesis pathway mostly occurs at the level of MS proteins.

## Conclusion

It is known since some time that regulatory redox switches control sulfur allocation between the primary and secondary routes of sulfur assimilation in Arabidopsis and other plants, notably under oxidative stress conditions during which there is an increased demand for reduced sulfur to support cysteine and glutathione synthesis. Accordingly, both APR and GCL in the primary metabolic pathway are activated upon oxidation whereas the activity of enzymes that are part of the secondary pathway, APK and SAL1, is attenuated. This results in directing APS into cysteine, methionine and glutathione synthesis and away from synthesis of PAPS. We provide here additional evidence, notably from redox proteomic approaches that most if not all enzymatic steps of the reductive sulfate assimilation and of methionine synthesis pathways may be controlled by oxidative modification of the enzymes, primarily protein persulfidation but also, disulfide bond formation, protein sulfenylation, nitrosylation, and glutathionylation to a lesser extent. These redox PTMs sometimes affect cysteines that are strictly conserved, at least in the photosynthetic organisms analyzed here, suggesting that the mechanism may be conserved and physiologically relevant in a wide range of species. However, they are also example of modifications that affect specific or different cysteine residues, notably in isoforms from angiosperms. This may indicate that additional layers of regulation exist as compared with cyanobacteria, microalgae or non-vascular plants as often observed for other metabolic pathways. It is obvious that identifying an oxidative modification and the targeted cysteine is insufficient and case-by-case studies have to be performed to decipher the impact on enzyme activity. The apparent prominent regulatory role of persulfidation may be consistent with the fact that H_2_S is produced by SIR in the frame of the reductive assimilation steps. Under oxidative conditions, sulfide could react with oxidized thiol groups, particularly sulfenic acids. Accordingly, a comparative study of sulfenylation and persulfidation reveals that sulfenylome and persulfidome overlap by approximately 80% (Aroca et al., 2021). Hence, persulfidation may represent a protective mechanism operating during oxidative stress conditions. Alternatively, it may constitute a regulatory mechanism that would help controlling protein function/activities in order to adjust the plant response to sulfate availability in case of oxidative stress (Wang et al., 2021). Incidentally, sulfide would be restituted upon persulfide reduction by a dithiol reductant.

## Author contributions

LB, ND, JC, and NR wrote sections of the manuscript. All authors contributed to the manuscript revision, read, and approved the submitted version.
