# Supplementary material for: Redox regulation of enzymes involved in sulfate assimilation and in the synthesis of sulfur-containing amino acids and glutathione in plants
Source: Front Plant Sci. 2022 Aug 16;13:958490. doi: 10.3389/fpls.2022.958490 (PMC9426629; doi:10.3389/fpls.2022.958490)
Supplement: Supplementary file 1 [file Data_Sheet_1.PDF]

Supplementary information to the paper "Redox regulation of enzymes involved in sulfate assimilation and in the synthesis of sulfur-containing amino acids and glutathione in plants" by de Bont Linda et al.

Redox post-translational modification	Method	Biological material	Protein hits	References
Persulfidation	Tag-Switch method followed by LC-MS/MS (identification) and tandem mass tag (quantification)	*Arabidopsis thaliana* leaves	2015 proteins	(Aroca et al., 2017)
	Tag-Switch method followed by LC-MS/MS	*Arabidopsis thaliana* roots	5214 proteins	(Jurado-Flores et al., 2021)
S-nitrosylation	Biotin switch: Purification of S-nitrosylated proteins by affinity chromatography on avidin column, 1D gel followed by LC-MS/MS & Purification of S-nitrosylated peptides by affinity chromatography followed by LC-MS/MS	*Arabidopsis thaliana* leaves	926 proteins	(Hu et al., 2015)
	Biotin switch: Purification of S-nitrosylated proteins by affinity chromatography (BST method) & Purification of S-nitrosylated peptides by affinity chromatography (SNOSID method) followed by LC-MS/MS	*Chlamydomonas reinhardtii*	492 proteins	(Morisse et al., 2014)
Glutathionylation	GSSG-biotin treatment followed by 2D-PAGE and MS analysis	*Arabidopsis thaliana* cell cultures	67 proteins	(Dixon et al., 2005)
S-cyanylation	2-imino-thiazolidine chemical method followed by 2D PAGE gels and mass spectrometry & direct untargeted analysis of proteins using LC-MS/MS	*Arabidopsis thaliana* leaves and roots	163 proteins	(García et al., 2019)
S-sulfenylation	Cys-SOH labeling with -(pent-4-yn-1-yl)-1H-benzo[c][1,2]thiazin-4(3H)-one 2,2-dioxide (BTD) probe followed by click chemistry of a biotin moiety, enrichment on streptavidin column and LC-MS/MS	*Arabidopsis thaliana* cell cultures	1394 proteins	(Huang et al., 2019)
	Expression of a genetically-encoded Yap1-cCRD construct fused to a tandem affinity purification (TAP) tag followed by enrichment on IgG-Sepharose beads and on an anti-YAP1C-derived peptide antibody and identification by LC-MS/MS	*Arabidopsis thaliana* cell cultures	>580 proteins	(Wei et al., 2020)
Reversible oxidation	Alkylation with N-ethylmaleimide, DTT reduction and biotin-HPDP tagging, trypsin digestion, affinity purification of the biotinylated peptides, labelling with different isotope-coded tags, identification and quantification by LC-MS/MS	*Arabidopsis thaliana* cell cultures	179 proteins	(Liu et al., 2014)

	Quantitative iodoacetyl tandem mass tag (iodoTMT)-based thiol redox proteomics	Citrate-respiring mitochondria of *Arabidopsis thaliana* seeds	425 proteins	(Nietzel et al., 2020)
TRX target	Affinity chromatography with a cytosolic *A. thaliana* TRXh3-C41S variant & reduction by TRXh3 followed by radioactive IAM labeling or biotinylated thiol reagent for avidin retention. These steps are followed by 2D gel separation and MS analysis	*Arabidopsis thaliana* leaves	73 proteins	(Marchand et al., 2006)
	Affinity chromatography with a cytosolic *A. thaliana* TRXh1-C43S variant followed by 2D gels and MS analysis	Whole cell extract of dark grown *Arabidopsis thaliana* plants	14 proteins	(Yamazaki et al., 2004)
	Affinity chromatography with a poplar TRXh1 C42S mutated variant & in vitro reduction by the NADPH/NTR-TRX system of a total soluble extracts separated by 2D gels, fluorescence labeling with monobromobimane (mBBr) and MS-MS analysis	*Triticum aestivum* endosperm and flour	68 proteins	(Wong et al., 2004)
	Affinity chromatography with a mitochondrial *A. thaliana* TRXo1-C121S variant followed by 2D gels and MS analysis	*Arabidopsis thaliana* mitochondria	101 proteins	(Yoshida et al., 2013)
	Alkylation of cysteine residues after DTT reduction, 1D gel coupled to MS analysis	Change in protein oxidation levels during dark to light transition of *Nicotiana tabacum* plants	27 proteins	(Zimmer et al., 2021)
	Affinity chromatography with a cytosolic *C. reinhardtii* TRXh1-C39S variant & in vitro reduction by cytosolic NADPH-NTR-CrTRXh1 system followed by LC-MS/MS	*Chlamydomonas reinhardtii*	1188 proteins	(Pérez-Pérez et al., 2017)
GRX target	Affinity chromatography with a poplar GRXC4 C30S mutated variant followed by LC-MS/MS	*Populus trichocarpa* & *A. thaliana* leaves, potato & *A. thaliana* leaf mitochondria, pea chloroplast	94 proteins	(Rouhier et al., 2005)

Table S1. Redox proteomic studies used to analyze the post-translational modifications of proteins involved in the sulfate assimilation pathway and in sulfur-containing amino acids and glutathione synthesis pathways.

Enzyme	AGI	Name	Subcellular localization	Cysteine position	Redox post-translational modification	References
ATP sulfurylase	At3g22890	ATPS1	Chloroplast	-	TRX target	(Marchand et al., 2006; Zimmer et al., 2021)
				-	Glutathionylation (Chlamydomonas)	(Zaffagnini et al., 2012)
				-	Nitrosylation (Chlamydomonas)	(Morisse et al., 2014)
				-	Persulfidation	(Aroca et al., 2017; Jurado-Flores et al., 2021)
				435	Nitrosylation	(Hu et al., 2015)
					Sulfenylation	(Wei et al., 2020)
	At1g19920	ATPS2	Chloroplast Cytosol	-	Glutathionylation	(Dixon et al., 2005)
				-	Persulfidation	(Aroca et al., 2017; Jurado-Flores et al., 2021)
	At4g14680	ATPS3	Chloroplast	-	-	-
	At5g43780	ATPS4	Chloroplast	-	Persulfidation	(Aroca et al., 2017; Jurado-Flores et al., 2021)
APS reductase	At4g04610	APR1	Chloroplast	-	Persulfidation	(Jurado-Flores et al., 2021)
	At1g62180	APR2	Chloroplast	-	Persulfidation	(Aroca et al., 2017; Jurado-Flores et al., 2021)
	At4g21990	APR3	Chloroplast	-	Persulfidation	(Aroca et al., 2017; Jurado-Flores et al., 2021)
Sulfite reductase	At5g04590	SIR	Chloroplast	-	Persulfidation	(Aroca et al., 2017; Jurado-Flores et al., 2021)
				-	TRX target	(Wong et al., 2004)
				245	Nitrosylation	(Hu et al., 2015)
O-acetylserine (thiol) lyase	At4g14880	OAS A1	Cytosol	-	Persulfidation	(Jurado-Flores et al., 2021)
				-	TRX target	(Marchand et al., 2006)
				42	Nitrosylation	(Hu et al., 2015)
					Reversible oxidation	(Liu et al., 2014)
					Sulfenylation	(Wei et al., 2020)
	At3g22460	OAS A2	Cytosol	45	Nitrosylation	(Hu et al., 2015)
					Reversible oxidation	(Liu et al., 2014)
					Sulfenylation	(Wei et al., 2020)
	At2g43750	OAS B	Chloroplast	-	Persulfidation	(Jurado-Flores et al., 2021)
				98	Reversible oxidation	(Liu et al., 2014)
					Sulfenylation	(Huang et al., 2019)
	At3g59760	OAS C	Mitochondrion	-	Persulfidation	(Jurado-Flores et al., 2021)
					TRX target<br>GRX target (poplar)	(Rouhier et al., 2005; Marchand et al., 2006; Yoshida et al., 2013)
				99 ; 136	Reversible oxidation	(Liu et al., 2014; Nietzel et al., 2020)
				150	Sulfenylation	(Huang et al., 2019)
Serine acetyltransferase	At5g56760	SERAT 1;1	Cytosol	-	Persulfidation	(Jurado-Flores et al., 2021)
	At1g55920	SERAT 2;1	Chloroplast	150	Sulfenylation	(Huang et al., 2019)
	At3g13110	SERAT 2;2	Mitochondrion	-	Persulfidation	(Jurado-Flores et al., 2021)
				227 ; 323	Sulfenylation	(Huang et al., 2019; Wei et al., 2020)
	At2g17640	SERAT 3;1	Cytosol	-	Persulfidation	(Jurado-Flores et al., 2021)
				122	Sulfenylation	(Huang et al., 2019)
	At4g35640	SERAT 3;2	Cytosol	159	Sulfenylation	(Huang et al., 2019)

Table S2. Redox post-translational modifications of enzymes involved in the primary sulfur assimilation pathway.

Enzyme	AGI	Name	Subcellular localization	Cysteine position	Redox post-translational modification	References
APS kinase	At2g14750	APK1	Chloroplast	-	Persulfidation	(Aroca et al., 2017; Jurado-Flores et al., 2021)
				-	Glutathionylation (Chlamydomonas)	(Pérez-Pérez et al., 2017)
				245	Nitrosylation	(Hu et al., 2015)
				86-119	Intermolecular disulfide	(Ravilious et al., 2012)
	At5g67520	APK2	Chloroplast Cytosol	-	-	-
	At3g03900	APK3	Chloroplast	-	-	-
	At4g39940	APK4	Chloroplast	-	Persulfidation	(Jurado-Flores et al., 2021)
Sulfotransferase	At5g43690	SOT1	Cytosol	-	-	-
	At3g51210	SOT2	Cytosol	-	-	-
	At4g26280	SOT3	Cytosol	-	-	-
	At2g27570	SOT4	Cytosol	-	-	-
	At3g45070	SOT5	Cytosol	-	Persulfidation	(Jurado-Flores et al., 2021)
	At3g45080	SOT6	Cytosol	-	-	-
	At1g28170	SOT7	Cytosol	-	-	-
	At1g13420	SOT8	Cytosol	-	Persulfidation	(Jurado-Flores et al., 2021)
	At1g13430	SOT9	Cytosol	-	-	-
	At2g14920	SOT10	Cytosol	-	-	-
	At2g03750	SOT11	Cytosol	-	Persulfidation	(Aroca et al., 2017)
	At2g03760	SOT12	Cytosol	-	Persulfidation	(Aroca et al., 2017; Jurado-Flores et al., 2021)
	At2g03770	SOT13	Cytosol	-	-	-
	At5g07000	SOT14	Cytosol	-	-	-
	At5g07010	SOT15	Cytosol	-	Persulfidation	(Jurado-Flores et al., 2021)
	At1g74100	SOT16	Cytosol	-	Persulfidation	(Aroca et al., 2017; Jurado-Flores et al., 2021)
	At1g18590	SOT17	Cytosol	-	Persulfidation	(Aroca et al., 2017; Jurado-Flores et al., 2021)
	At1g74090	SOT18	Cytosol	-	Persulfidation	(Aroca et al., 2017; Jurado-Flores et al., 2021)
Phosphatase	At5g63980	SAL1	Chloroplast Mitochondrion	-	Persulfidation	(Aroca et al., 2017; Jurado-Flores et al., 2021)
				119	Nitrosylation	(Hu et al., 2015)
				119	Reversible oxidation	(Liu et al., 2014)
				119	Intermolecular disulfide	(Chan et al., 2016)
				67-190	Intramolecular disulfide	(Chan et al., 2016)

Table S3. Redox post-translational modifications of enzymes involved in the secondary sulfur metabolic pathway.

Enzyme	AGI	Name	Subcellular localization	Cysteine position	Redox post-translational modification	References
Glutathione reductase	At3g24170	GR1	Cytosol Peroxisome	-	Persulfidation	(Aroca et al., 2017; Jurado-Flores et al., 2021)
				73	Nitrosylation	(Begara-Morales et al., 2015; Hu et al., 2015)
	At3g54660	GR2	Chloroplast Mitochondrion	-	GRX target	Rouhier et al., 2005
				-	Persulfidation	(Aroca et al., 2017; Jurado-Flores et al., 2021)
Glutathione synthase	At4g23100	GCL/GSH1	Chloroplast	-	Persulfidation	(Aroca et al., 2017; Jurado-Flores et al., 2021)
				349	Nitrosylation	(Hu et al., 2015)
				349	Reversible oxidation	(Liu et al., 2014)
				186-406	Intramolecular disulfide	(Hicks et al., 2007)
				349-364	Intramolecular disulfide	(Hicks et al., 2007)
				349	Sulfenylation	(Wei et al., 2020)
	At5g27380	GSH2	Chloroplast Cytosol	-	Persulfidation	(Aroca et al., 2017; Jurado-Flores et al., 2021)
				134	Nitrosylation	(Hu et al., 2015)
					Sulfenylation	(Wei et al., 2020)

Table S4. Redox post-translational modifications of enzymes involved in the glutathione biosynthesis pathway.

Enzyme	AGI	Name	Subcellular localization	Cysteine position	Redox post-translational modification	References
Cystathionine γ-synthase	At3g01120	CGS	Chloroplast	334	Sulfenylation	(Wei et al., 2020)
					Cysteine reversible oxidation	(Liu et al., 2014)
Cystathionine β-lyase	At3g57050	CBL	Chloroplast	-	Persulfidation	(Jurado-Flores et al., 2021)
				417	Nitrosylation	(Hu et al., 2015)
Methionine synthase	At5g17920<br>At3g03780	MS1<br>MS2	Cytosol	-	Glutathionylation	(Dixon et al., 2005)
				-	Persulfidation	(Jurado-Flores et al., 2021)
				-	TRX target<br>(Arabidopsis, Chlamydomonas)<br>GRX target (poplar)	(Yamazaki et al., 2004; Pérez-Pérez et al., 2017)
				328	Sulfenylation	(Wei et al., 2020)
					Nitrosylation	(Hu et al., 2015)
				522	Sulfenylation	(Huang et al., 2019; Wei et al., 2020)
					Nitrosylation	(Hu et al., 2015)
				580<br>733	S-cyanylation	(García et al., 2019)
					Sulfenylation	(Huang et al., 2019; Wei et al., 2020)
					Sulfenylation	(Wei et al., 2020)
	At5g20980	MS3	Chloroplast	-	TRX target (wheat, Chlamydomonas)<br>GRX target (poplar)	(Wong et al., 2004; Rouhier et al., 2005; Pérez-Pérez et al., 2017)
				-	Glutathionylation (Chlamydomonas)	(Zaffagnini et al., 2012)
				-	Nitrosylation (Chlamydomonas)	(Morisse et al., 2014)

Table S5. Redox post-translational modifications of enzymes involved in the methionine biosynthesis pathway.

References

Aroca, A., Benito, J. M., Gotor, C., and Romero, L. C. (2017). Persulfidation proteome reveals the regulation of protein function by hydrogen sulfide in diverse biological processes in Arabidopsis. *Journal of Experimental Botany* 68, 4915–4927. doi: 10/gcfww3.

Begara-Morales, J. C., Sánchez-Calvo, B., Chaki, M., Mata-Pérez, C., Valderrama, R., Padilla, M. N., et al. (2015). Differential molecular response of monodehydroascorbate reductase and glutathione reductase by nitration and S-nitrosylation. *Journal of Experimental Botany* 66, 5983–5996. doi: 10/f7s5bb.

Chan, K. X., Mabbitt, P. D., Phua, S. Y., Mueller, J. W., Nisar, N., Gigolashvili, T., et al. (2016). Sensing and signaling of oxidative stress in chloroplasts by inactivation of the SAL1 phosphoadenosine phosphatase. *Proc. Natl. Acad. Sci. U.S.A.* 113, E4567-4576. doi: 10.1073/pnas.1604936113.

Dixon, D. P., Skipsey, M., Grundy, N. M., and Edwards, R. (2005). Stress-induced protein S-glutathionylation in Arabidopsis. *Plant Physiol* 138, 2233–2244. doi: 10/d9jz9g.

García, I., Arenas-Alfonseca, L., Moreno, I., Gotor, C., and Romero, L. C. (2019). HCN Regulates Cellular Processes through Posttranslational Modification of Proteins by S-cyanylation. *Plant Physiol* 179, 107–123. doi: 10.1104/pp.18.01083.

Hicks, L. M., Cahoon, R. E., Bonner, E. R., Rivard, R. S., Sheffield, J., and Jez, J. M. (2007). Thiol-Based Regulation of Redox-Active Glutamate-Cysteine Ligase from *Arabidopsis thaliana*. *The Plant Cell* 19, 2653–2661. doi: 10/dmv9nx.

Hu, J., Huang, X., Chen, L., Sun, X., Lu, C., Zhang, L., et al. (2015). Site-Specific Nitrosoproteomic Identification of Endogenously S-Nitrosylated Proteins in Arabidopsis. *Plant Physiol* 167, 1731–1746. doi: 10/f7bmsh.

Huang, J., Willems, P., Wei, B., Tian, C., Ferreira, R. B., Bodra, N., et al. (2019). Mining for protein S-sulfenylation in Arabidopsis uncovers redox-sensitive sites. *Proc. Natl. Acad. Sci. U.S.A.* 116, 21256–21261. doi: 10/gnjkhg.

Jurado-Flores, A., Romero, L. C., and Gotor, C. (2021). Label-Free Quantitative Proteomic Analysis of Nitrogen Starvation in Arabidopsis Root Reveals New Aspects of H2S Signaling by Protein Persulfidation. *Antioxidants (Basel, Switzerland)* 10, 508. doi: 10/gphk95.

Liu, P., Zhang, H., Wang, H., and Xia, Y. (2014). Identification of redox-sensitive cysteines in the Arabidopsis proteome using OxiTRAQ, a quantitative redox proteomics method. *Proteomics* 14, 750–762. doi: 10/f2qg26.

Marchand, C., Le Maréchal, P., Meyer, Y., and Decottignies, P. (2006). Comparative proteomic approaches for the isolation of proteins interacting with thioredoxin. *Proteomics* 6, 6528–6537. doi: 10/c5x6pp.

Morisse, S., Zaffagnini, M., Gao, X.-H., Lemaire, S. D., and Marchand, C. H. (2014). Insight into protein S-nitrosylation in *Chlamydomonas reinhardtii*. *Antioxid Redox Signal* 21, 1271–1284. doi: 10/f6ghmw.

Nietzel, T., Mostertz, J., Ruberti, C., Née, G., Fuchs, P., Wagner, S., et al. (2020). Redox-mediated kick-start of mitochondrial energy metabolism drives resource-efficient seed germination. *Proc. Natl. Acad. Sci. U.S.A.* 117, 741–751. doi: 10.1073/pnas.1910501117.

Pérez-Pérez, M. E., Mauriès, A., Maes, A., Tourasse, N. J., Hamon, M., Lemaire, S. D., et al. (2017). The Deep Thioredoxome in *Chlamydomonas reinhardtii*: New Insights into Redox Regulation. *Molecular Plant* 10, 1107–1125. doi: 10/gbsft2.

Ravilious, G. E., Nguyen, A., Francois, J. A., and Jez, J. M. (2012). Structural basis and evolution of redox regulation in plant adenosine-5′-phosphosulfate kinase. *Proceedings of the National Academy of Sciences U.S.A.* 109, 309–314. doi: 10/fzh6h5.

Rouhier, N., Villarejo, A., Srivastava, M., Gelhaye, E., Keech, O., Droux, M., et al. (2005). Identification of plant glutaredoxin targets. *Antioxid Redox Signal* 7, 919–929. doi: 10.1089/ars.2005.7.919.

Wei, B., Willems, P., Huang, J., Tian, C., Yang, J., Messens, J., et al. (2020). Identification of Sulfenylated Cysteines in *Arabidopsis thaliana* Proteins Using a Disulfide-Linked Peptide Reporter. *Frontiers in Plant Science* 11. doi: 10/gphk94.

Wong, J. H., Cai, N., Balmer, Y., Tanaka, C. K., Vensel, W. H., Hurkman, W. J., et al. (2004). Thioredoxin targets of developing wheat seeds identified by complementary proteomic approaches. *Phytochemistry* 65, 1629–1640. doi: 10/ffcgqb.

Yamazaki, D., Motohashi, K., Kasama, T., Hara, Y., and Hisabori, T. (2004). Target proteins of the cytosolic thioredoxins in *Arabidopsis thaliana*. *Plant Cell Physiol* 45, 18–27. doi: 10.1093/pcp/pch019.

Yoshida, K., Noguchi, K., Motohashi, K., and Hisabori, T. (2013). Systematic exploration of thioredoxin target proteins in plant mitochondria. *Plant Cell Physiol.* 54, 875–892. doi: 10.1093/pcp/pct037.

Zaffagnini, M., Bedhomme, M., Marchand, C. H., Morisse, S., Trost, P., and Lemaire, S. D. (2012). Redox regulation in photosynthetic organisms: focus on glutathionylation. *Antioxid Redox Signal* 16, 567–586. doi: 10.1089/ars.2011.4255.

Zimmer, D., Swart, C., Graf, A., Arrivault, S., Tillich, M., Proost, S., et al. (2021). of the redox network during induction of photosynTopology thesis as revealed by time-resolved proteomics in tobacco. *Science Advances* 7, eabi8307. doi: 10/gphk93.
